# Infliximab in young paediatric IBD patients: it is all about the dosing

**DOI:** 10.1007/s00431-020-03750-0

**Published:** 2020-08-19

**Authors:** Maria M. E. Jongsma, Dwight A. Winter, Hien Q. Huynh, Lorenzo Norsa, Seamus Hussey, Kaija-Leena Kolho, Jiri Bronsky, Amit Assa, Shlomi Cohen, Raffi Lev-Tzion, Stephanie Van Biervliet, Dimitris Rizopoulos, Tim G. J. de Meij, Dror S. Shouval, Eytan Wine, Victorien M. Wolters, Christine Martinez-Vinson, Lissy de Ridder, on behalf of the Paediatric IBD Porto Group of ESPGHAN

**Affiliations:** 1 Department of Paediatric Gastroenterology, Erasmus Medical Center/Sophia Children’s Hospital, Rotterdam, The Netherlands; 2 Division of Paediatric Gastroenterology and Nutrition, Edmonton Paediatric IBD Clinic (EPIC), University of Alberta, Edmonton, Canada; 3 Department of Paediatric Gastroenterology Hepatology and Nutrition, Hôpital Necker-Enfants-Malades, Paris, France; 4 Department of Paediatric Gastroenterology, Our Lady’s Children’s Hospital and RCSI, Dublin, Ireland; 5 Department of Paediatric Gastroenterology, Tampere University Hospital and University of Tampere, Tampere, Finland and Children’s Hospital, University of Helsinki, Helsinki, Finland; 6 Department of Paediatrics, Gastroenterology and Nutrition Unit, University Hospital Motol, Prague, Czech Republic; 7 Institute of Paediatric Gastroenterology, Schneider Children’s Hospital, affiliated to the Sackler Faculty of Medicine, Tel Aviv University, Tel Aviv, Israel; 8 Paediatric Gastroenterology unit, Dana-Dwek Children’s Hospital, Tel Aviv Sourasky Medical Center and the Sackler Faculty of Medicine, Tel Aviv University, Tel Aviv, Israel; 9 Department of Paediatric Gastroenterology, Shaare Zedek Medical Center, Jerusalem, Israel; 10 Department of Paediatric Gastroenterology, Universitair Ziekenhuis, Ghent, Belgium; 11 Department of Biostatistics, Erasmus Medical Center, Rotterdam, The Netherlands; 12 Department of Paediatric Gastroenterology, Amsterdam UMC - Vrije Universiteit/Emma Children’s Hospital, Amsterdam, The Netherlands; 13 Paediatric Gastroenterology Unit, Edmond and Lily Safra Children’s Hospital Sheba Medical Center, Ramat Gan, and Sackler Faculty of Medicine, Tel Aviv University, Tel Aviv, Israel; 14 Department of Paediatric Gastroenterology, Utrecht Medical Center/Wilhelmina Children’s Hospital, Utrecht, The Netherlands; 15 Department of Paediatric Gastroenterology, Hôpital Robert Debré, Paris, France

**Keywords:** Crohn’s disease, Ulcerative colitis, Anti-TNF, Clinical pharmacology, Paediatric, Gastroenterology, Biologics

## Abstract

Infliximab (IFX) is administered intravenously using weight-based dosing (5 mg/kg) in inflammatory bowel disease (IBD) patients. Our hypothesis is that especially young children need a more intensive treatment regimen than the current weight-based dose administration. We aimed to assess IFX pharmacokinetics (PK), based on existing therapeutic drug monitoring (TDM) data in IBD patients < 10 years. TDM data were collected retrospectively in 14 centres. Children treated with IFX were included if IFX was started as IBD treatment at age < 10 years (young patients, YP) and PK data were available. Older IBD patients aged 10–18 years were used as controls (older patients, OP). Two hundred and fifteen paediatric inflammatory bowel disease (PIBD) patients were eligible for the study (110 < 10 year; 105 ≥ 10 years). Median age was 8.3 years (IQR 6.9–8.9) in YP compared with 14.3 years (IQR 12.8–15.6) in OP at the start of IFX. At the start of maintenance treatment, 72% of YP had trough levels below therapeutic range (< 5.4 μg/mL). After 1 year of scheduled IFX maintenance treatment, YP required a significantly higher dose per 8 weeks compared with OP (YP; 9.0 mg/kg (IQR 5.0–12.9) vs. OP; 5.5 mg/kg (IQR 5.0–9.3); *p* < 0.001). The chance to develop antibodies to infliximab was relatively lower in OP than YP (0.329 (95% CI − 1.2 to − 1.01); *p* < 0.001), while the overall duration of response to IFX was not significantly different (after 2 years 53% (*n* = 29) in YP vs. 58% (*n* = 45) in OP; *p* = 0.56).

*Conclusion*: Intensification of the induction scheme is suggested for PIBD patients aged < 10 years.

**Table:** 

*What is Known?*	
•Infliximab trough levels of paediatric IBD patients are influenced by several factors as dosing scheme, antibodies and inflammatory markers. •In 4.5–30% of the paediatric IBD patients, infliximab treatment was stopped within the first year.	
*What is New?*	
•The majority of young PIBD (< 10 years) have inadequate IFX trough levels at the start of maintenance treatment. •Young PIBD patients (< 10 years) were in need of a more intensive treatment regimen compared with older paediatric patients during 1 year of IFX treatment. •The chance to develop antibodies to infliximab was relatively higher in young PIBD patients (< 10 years).

**Electronic supplementary material:**

The online version of this article (10.1007/s00431-020-03750-0) contains supplementary material, which is available to authorized users.

## Introduction

Treatment options for inflammatory bowel disease (IBD) radically changed after the introduction of monoclonal antibodies to tumour necrosis factor alpha (TNF-α). Infliximab (IFX), the first developed anti-TNF-α biological agent [1], is administered intravenously in a weight-based dose (5 mg/kg) in both paediatric and adult patients during induction (infusion at weeks 0, 2 and 6) and maintenance phase (every 8 weeks) [2].

Contradictory to this dosing scheme, Dotan et al. showed a nonlinear correlation between higher body weight and IFX clearance. Therefore, in lower body weight patients, it would be expected to have a lower drug exposure at all times than patients with a higher body weight after administration of 5 mg/kg [3]. In paediatric patients, a 25–40% lower drug exposure was found compared with adults [4, 5]. This implies that paediatric patients with low body weight are more likely to be underdosed.

However, even within the paediatric IBD (PIBD) population, response rates may differ significantly [4, 6–8]. Kelsen et al. [9] found that paediatric CD patients 7 years or younger showed lower IFX response rates and were less likely to continue IFX therapy compared with older paediatric CD patients [10].

Besides weight, developmental changes in body composition are known to affect absorption, distribution, metabolism and excretion of a biological agent which explains a larger variability of IFX disposition [4, 11, 12]. Moreover, pharmacokinetic (PK) population studies in older paediatric and adult IBD patients show that inflammatory burden, serum albumin levels, concomitant immunomodulators and presence of antibodies to infliximab (ATIs) [3, 13, 14] affect drug clearance.

However, data on IFX trough levels within this special population are still limited. For this reason, the primary aim of this study was to investigate the pharmacological response to IFX, based on existing therapeutic drug monitoring (TDM) data, in young (< 10 years) PIBD patients and to compare these with data of older (≥ 10 years) PIBD patients.

## Methods

### Study design and patient management

In this retrospective case-controlled study, data of children treated with IFX between 2004 and 2019 were collected between 2015 and 2019 from centres in Europe and Canada as a PIBD Porto group of ESPGHAN initiative. For the young patient (YP) group, PIBD patients were eligible if IFX treatment was started for active disease before age of 10 years. These data were compared with a control group of paediatric IFX treated IBD patients above age of 10. This cut-off was based on age groups classified by the Paris classification [15]. OP were selected based on the time period IFX treatment was started. Patients were excluded if data on IFX dosing or levels were missing or if a patient was diagnosed with a monogenetic disease. Investigators from participating centres were asked to enter all patient information into Castor, an electronic data capturing tool (Amsterdam, the Netherlands). Recorded anthropometric and longitudinal data on clinical parameters and PK/pharmacodynamic (PD) parameters, of YP (< 10 years at time of IFX initiation) and older patients (OP; ≥ 10 years at time of IFX initiation), were compared. IFX trough levels could be collected pro- or reactively.

### Outcome measures and definitions

Clinical disease activity was defined by Physician Global Assessment (PGA) scores. Quiescent disease and clinical remission were defined as 0, if in the last week the patient had minimal or no symptoms thought to be secondary to IBD, mild disease as 1 (mild recurring or persistent symptoms thought to be secondary to IBD), moderate disease as 2 (moderate or combinations of mild and moderate recurring or persistent symptoms secondary to IBD) and severe as 3 (severe or combinations of moderate and severe recurring or persistent symptoms thought to be due to IBD) [16]. Drug levels were defined as subtherapeutic if IFX serum trough levels were < 29 μg/mL at 2 weeks, < 18 μg/mL at 6 weeks [17] and < 5.4 μg/mL at 14 weeks after start IFX treatment [18, 19]. A second reference cut-off < 3.1 μg/mL for maintenance treatment in adult patients was used to compare our paediatric patients with adult patients [14, 20]. Treatment intensification by dose intensification and/or interval adjustment was done based on physician’s discretion. Loss of response due to PK was defined as having low trough levels despite adequate dosing. Loss of response due to pharmacodynamics was defined as loss of response to therapy despite adequate trough levels (trough level ≥ 5.4 μg/mL). Loss of response due to immunogenicity was defined as having low trough levels and proven presence of ATI. Primary nonresponse was defined as equal or increased PGA score after completion of induction therapy (14 weeks). Secondary loss of response was defined as recurring symptoms after initial clinical benefit (increased PGA score) resulting in treatment change [21]. ATIs were defined as positive if antibodies were measured above the cut-off point of the specific assays used (Online Resource 1). In all centres, ESR levels were determined with the Westgren method.

Primary outcome was the comparison of dosing and treatment intervals between age groups after 1 year of IFX treatment. Secondary outcomes were the following: (1) Evaluation of trough levels during induction (weeks 2 and 6) and maintenance treatment (weeks 14, 52 and 104) in YP with proactively collected trough levels, (2) Primary nonresponse to IFX treatment, (3) Clinical remission 1 year after start of IFX treatment, (4) Identification of independent clinical predictors influencing IFX trough levels, (5) ATI development within 52 weeks, (6) IFX treatment failure at 52 weeks.

### Statistical analysis

Continuous variables, normally distributed, were reported as means and standard deviations and compared with the *t* test. Continuous variables not following normal distribution were analysed by the Mann-Whitney *U* test and presented as medians and interquartile range (IQR). Ordinal variables were analysed by the Kruskal-Wallis test. Categorical variables were presented as absolute frequencies and percentages and compared by the *χ*^2^ test or Fisher exact test. Three different linear mixed models were constructed, the first to evaluate trough levels during induction and maintenance treatment, the second to describe the evolution of trough levels over time and identify independent predictors affecting this evolution and a third model to evaluate differences in ATI levels (Online Resource 2). A Kaplan-Meier survival analysis was performed to evaluate duration of IFX treatment. The significance level was set to 0.05; no corrections were performed for multiple testing. Calculations were performed using IBM SPSS Statistics 24.0 (IBM Corp, Armonk, NY).

## Results

### Baseline characteristics

Two hundred and fifteen PIBD patients were enrolled in the study, 110 young IBD patients (< 10 years), whereof 14/110 were very early-onset inflammatory bowel disease (VEO-IBD) patients (< 6 years), and 105 older IBD patients (≥ 10 and < 18 years) at initiation of IFX treatment. Median age at the start of IFX treatment was 8.3 (IQR 6.9–8.9) years in YP, while this was 14.3 (IQR 12.8–15.6) years in OP. In the YP group, fewer patients (66/110, 60%) were diagnosed with CD, compared with the OP group (81/105, 77%); *p* = 0.024). Inflammatory markers and disease location were comparable in both age groups at the start of IFX treatment (Table 1)*.* Despite IFX was started with a median dose of 5 mg/kg (IQR5-5) in both groups, YP at start received a significant higher dose (*p* = 0.015). Seventy-seven percent (85/110) of YP and 90% (95/105) of OP received 5 mg/kg. In 11% (12/110) of YP, already a dose of ≥ 8 mg/kg was started, while this was done in 3% (3/105) of OP. YP were included in 14 different centres (median 6 [IQR 4–8] patients per centre), and OP were included in 9 centres (median 10 [IQR-3-10]).

**Table 1:** Baseline characteristics of patients at start infliximab treatment

			Total, *n* = 215	YP start IFX < 10 years of age, *n* = 110	OP start IFX ≥ 10 years of age, *n* = 105	*p* value
Age at diagnosis in years (IQR)			9.22 (6.6–12.9)	6.71 (5.12–8.36)	12.93 (11.65–14.55)	*< 0.001*
Age at start treatment in years (IQR)			9.72 (8.26–14.0)	8.32 (6.95–8.93)	14.32 (12.79–15.61)	*< 0.001*
Sex (%)	Male		122 (57%)	58 (53%)	64 (61%)	0.224
BSA (IQR)	*n* = 171		1.53 (0.90–1.48)	0.90 (0.78–1.02).	1.52 (1.29–1.69)	*< 0.001*
Diagnosis (%)	Crohn’s disease		147 (69%)	66 (60%)	81 (77%)	*0.024*
	Ulcerative colitis		50 (23%)	33 (30%)	17 (16%)	
	IBD unclassified		18 (8%)	11 (10%)	7 (7%)	
Ethnicity (%)	Afro-Caribbean		19 (9%)	8 (7%)	10 (10%)	0.388
	Arab		33 (15%)	14 (12.8%)	19 (18%)	
	Asian		1 (1%)	1 (0.9%)	0 (0%)	
	Caucasian		149 (70%)	79 (73%)	72 (69%)	
	Other		11 (5%)	8 (7.3%)	3 (3%)	
TPMT status (%)	Homozygous		78 (36%)	46 (42%)	32 (30%)	0.295
	Heterozygous		11 (5%)	4 (4%)	7 (7%)	
	Unknown		126 (59%)	60 (54%)	66 (63%)	
ESR (mm/h) (IQR)	*n* = 161		28 (19–42)	30 (20–42)	27 (16–49)	0.654
CRP (mg/L) (IQR)	*n* = 180		11 (3.4–40.5)	10 (3–25)	20 (4–52)	0.066
Albumin (g/L) (IQR)	*n* = 177		37 (33–42)	35 (31.2–39.6)	40 (34–43)	0.095
Clinical disease activity (%)	Quiescent 0		4 (2%)	3 (3%)	1 (1%)	0.659
	Mild 1		15 (11%)	11 (13%)	8 (12%)	
	Moderate 2		80 (37%)	41 (48%)	38 (47%)	
	Severe 3		65 (30%)	31 (36%)	34 (42%)	
Median start dose (mg/kg) (IQR)			5 (5–5)	5 (5–5)	5 (5–5)	*0.015*
Paris classification	Age	A1a	115 (54%)	110 (100%)	5 (5%)	*< 0.001*
		A1b	100 (46%)		100 (95%)	
For CD patients	Location	L1	15 (13%)	7 (14%)	8 (11%)	0.071
		L2	37 (31%)	21 (43%)	16 (23%)	
		L3	68 (56%)	21 (43%)	45 (65%)	
		L4a	31 (26%)	14 (21%)	17 (21%)	
		L4b	5 (4%)	3 (5%)	2 (3%)	
	Behaviour	B1	100 (83%)	38 (76%)	60 (87%)	0.126
		B2	11 (9%)	5 (10%)	6 (9%)	
		B3	7 (5%)	6 (12%)	1 (1%)	
		B2B3	3 (2%)	1 (2%)	2 (3%)	
	Perianal disease (%)		43 (36%)	22 (43%)	21 (31%)	
	Growth delay (%)		43 (37%)	19 (37%)	24 (36%)	
For UC and IBD-U patients	Extent	1	1 (2%)	–	1 (5%)	0.747
		2	4 (9%)	2 (9%)	2 (8%)	
		3	5 (11%)	3 (14%)	2 (8%)	
		4	36 (78%)	17 (77%)	19 (79%)	
	Ever severe (%)		38 (83%)	17 (79%)	21 (88%)	0.361

*p* values are from Fisher’s exact test for categorical variables and from Kruskal-Wallis or Mann-Whitney *U* test for continuous variables. *p* values < 0.05 were considered as significant

*Abbreviations*: *YP* young patients, *OP* older patients, *TPMT* thiopurine methyltransferase, *ESR* erythrocyte sedimentation, *CRP* C-reactive protein, *BSA* body surface area, *IQR* interquartile range, % percentage

In multivariate analyses, IFX trough levels were not significantly different between UC and CD patients; thus, the data of patients with both diagnoses are pooled (*β* − 0.29; 95% CI − 0.640 to 0.059, *p* = 0.101); Table 2). In addition, a separate analysis for UC and CD patients is shown in Table 3.

**Table 2:** Predictors of infliximab trough level

	*B*	*p* value	95% CI
Intercept	0.419	0.317	(− 0.410 to 1.249)
Reactive sample collection	− 0.235	0.345	(− 0.672 to 0.034)
Time in days	< − 0.001	0.767	(− 0.003 to < 0.001)
Male sex	− 0.024	0.882	(− 0.351 to 0.303)
Diagnose CD	− 0.291	0.101	(− 0.640 to 0.059)
Age at diagnosis	− 0.013	0.613	(− 0.063 to 0.038)
Albumin (g/L)	< − 0.001	0.938	(− 0.011 to 0.012)
CRP (mg/L)	0.003	0.379	(− 0.011 to 0.004)
ESR (mm/h)	− 0.004	0.270	(− 0.012 to < 0.004)
Clinical disease activity	− 0.014	0.845	(− 0.153 to 0.125)
ATI positive	− 0.681	*< 0.001*	(0.446 to 0.914)
Immunomodulator use	− 0.149	0.140	(− 0.348 to 0.049)
Dose (mg/kg)	0.050	0.051	(< 0.001 to 0.100)
Interval (days)	− 0.006	0*.011*	(− 0.010 to − 0.001)

Linear mixed model analysis is performed to investigate the influence of different predictors on IFX trough levels. *p* values < 0.05 were considered as significant

*Abbreviations*: *B* beta, *sig.* significant, *CI* confidence interval, *CRP* C-reactive protein, *BSA* body surface area, *ESR* erythrocyte sedimentation rate, *IFX* infliximab, *ATI* antibody to infliximab

**Table 3:** Outcome measures split out for diagnosis CD and UC/IBDU. (A) Proportion of patients with treatment changes. (B) Median trough levels (IQR) during induction and maintenance treatment at 2, 6, 14 and 52 weeks

**(A)**						
**Outcome measures**	**Young CD patients**	**Young UC/IBDU patients**	***p*** **value**	**Older CD patients**	**Older UC /IBDU patients**	***p*** **value**
Primary nonresponse, *n* (%)	6/66 (9%)	6/42 (14%)	0.402	2/75 (3%)	3/20 (15%)	*0.028*
Outcome measures at 52 weeks						
Received treatment escalation, *n* (%)	33/45 (73%)	18/24 (75%)	0.881	27/61 (44%)	6/12 (50%)	0.715
Dose intensification, *n* (%)	27/45 (39%)	15/24 (63%)	0.839	16/61 (29%)	6/12 (50%)	0.101
Interval adjustment, *n* (%)	28/45 (62%)	11/23 (48%)	0.256	20/62 (32%)	3/12 (25%)	0.619
Clinical remission with IFX, *n* (%)	19/57 (33%)	12/37 (32%)	0.928	27/58 (47%)	7/21 (33%)	0.295
**(B)**						
**Median trough levels during induction and maintenance treatment**		**Young CD patients**		**Young UC/IBDU patients**	***p*** **value**	
Trough levels at week 2 μg/mL (IQR)		10.1 (7.9–16.1) (*n* = 21)		15.2 (10.4–18.8) (*n* = 9)	0.808	
Trough levels at week 6 μg/mL (IQR)		7.2 (3.8–12.7) (*n* = 23)		10.4 (0.75–12.5) (*n* = 14)	0.487	
Trough levels at week 14 μg/mL (IQR)		3.1 (1–6.0) (*n* = 25)		2.9 (0.0–11.5) (*n* = 10)	0.584	
Trough levels at week 52 μg/mL (IQR)		6.4 (2.1–10.8) (*n* = 13)		1.2 (0.0–1.9) (*n* = 5)	*0.035*	

*p* values < 0.05 were considered as significant

*Abbreviations*: *CD* Crohn’s disease, *UC* ulcerative colitis, *IFX* infliximab, *IQR* Inter quartile range

### YP received a more intensive IFX treatment schedule compared with OP after 1 year of IFX

In the YP group, 12/110 (11%) showed primary nonresponse to IFX treatment compared with 5/105 (5%) OP (*p* = 0.133). In these patients, IFX already was discontinued during induction in a comparable number of 9/110 (8%) YP and 3/105 (3%) OP (*p* = 0.09). In YP, IFX was discontinued because of immunogenicity (*n* = 3), PK (*n* = 2), PD (*n* = 2) or other reasons (*n* = 2), while in OP, this was related to pharmacodynamics in all cases (*n* = 3).

After 1 year of scheduled IFX maintenance treatment, YP required a significantly higher dose per 8 weeks compared with OP (YP; 9.0 mg/kg (IQR 5.0–12.9) vs. OP; 5.5 mg/kg (IQR 5.0–9.3); *p* < 0.001). This also was reflected in the number of patients on IFX receiving treatment escalation, 52/71 (73%) YP vs. 34/76 (45%) OP, *p* < 0.001 (Table 4).

**Table 4:** Treatment strategy by 1 year after start of infliximab

	YP start IFX < 10 years of age	OP start IFX ≥ 10 years of age	*p* value
Patients on IFX at 52 weeks (*n*)	71/94	76/95	
Patients received treatment escalation, *n* (%)	52/71 (73%)	34/76 (45%)	*< 0.001*
Dose intensification, *n* (%)	42/71 (61%)	24/76 (32%)	*0.001*
Interval adjustment, *n* (%)	39/71 (57%)	24/76 (32%)	*0.002*
Mg/kg per 8 week interval at 52 weeks; median (IQR)	9.0 (5–12.9)	5.5 (5–9.3)	*< 0.001*
Median interval in days (IQR)	49 (39–56)	56 (49–57)	*0.002*
Median dose (mg/kg) (IQR) (not corrected for interval in weeks)	8 (5–10)	5 (5–8)	*0.002*
Patients lost to follow-up at week 52	94/110 (15%)	95/105 (10%)	

Primary nonresponse IFX was defined as equal or increased PGA score after completion of induction therapy (14 weeks). *p* values < 0.05 were considered as significant

*Abbreviation*: *YP* young patients, *OP* older patients, *IQR* interquartile range, *%* percentage

### Clinical remission at 1 year

In 94/110 (85%) YP and 79/105 (75%) OP, data of clinical remission rate after 1 year were available. The number of patients in clinical remission while still on IFX maintenance was comparable (31/94 (33%) in YP vs. 34/79 (43%) in OP; *p* = 0.174) for both groups after 1 year. However, within this subgroup, significantly less YP were still receiving a weight based 5 mg/kg IFX treatment scheduled every 8 weeks (8/31 (26%) of YP compared with 20/33 (59%) of OP; *p* = 0.007). The proportion of YP patients in clinical remission was similar in YP with a high dose at start (> 8 mg/kg) (3/11 (27%)) compared with patients started with standard dose (≤ 5 mg/kg; 22/69 (32%); *p* = 0.759). Proactively collected trough levels did not result in a higher proportion of patients in clinical remission. Ten of 44 patients with proactive collection were in clinical remission at week 52 while this was the case for 20/50 patients with reactively determined trough levels after start IFX, *p* = 0.123.

### IFX trough levels are suboptimal at the start of maintenance treatment in the majority of YP

In a subgroup (46/110) of YP, 414 trough levels were proactively determined during follow-up; an overview of these trough levels is shown in Table 5. At the start of maintenance treatment (week 14), the median trough level in this group was 3.1 μg/mL (IQR 1–6.4). The percentage of YP with subtherapeutic trough levels (< 5.4 μg/mL) was 72% (25/35). Even according the lower target levels in adult literature, still 17/35 (49%) patients had a subtherapeutic level (< 3.1 μg/mL). YP with a trough level < 5.4 μg/mL at week 14 significantly more often developed ATI within the first year (16/18 patients), compared with YP with trough levels ≥ 5.4 μg/mL at week 14 (8/17; *p* = 0.02). Out of the 917 IFX trough levels (proactive and reactive) analysed in the whole cohort, 109 samples were measured during IFX induction. Combined mean trough levels of weeks 2 and 6 were comparable in YP (*n* = 86) compared with OP (exp *β* − 0.15, 95% CI − 0.44 to 0.15; *p* = 0.323), although the number of levels drawn in OP (*n* = 23) is low. During maintenance treatment, mean IFX trough levels in YP (*n* = 536) and OP (*n* = 272) were similar (exp *β* − .11, 95% CI − 0.52 to 0.30; *p* = 0.593).

**Table 5:** Infliximab trough levels routinely measured in young patients

	Median dose (mg/kg)	Trough level (μg/mL) (IQR)	Recommended TL level	% below recommended level	% of measured patients in remission
IFX week 2 (*n* = 30)	5 mg/kg (IQR 5–5)	12.5 μg/mL (8.2–17.5)	29 μg/mL^†^	97%	27%
IFX week 6 (*n* = 37)	5 mg/kg (IQR 5–5.1)	8.2 μg/mL (3.1–12.5)	18 μg/mL^†^	87%	40%
IFX week 14 (*n* = 35)	5 mg/kg (IQR 5–8.7)	3.1 μg/mL (1–6.4)	5.4 μg/mL^‡^	72%	43%
52 weeks (*n* = 18)	7.5 mg/kg (IQR 5–10)	4.4 μg/mL (0.8–6.3)	5.4 μg/mL^‡^	50%	42%
104 weeks (*n* = 8)	6.5 mg/kg (IQR 5–10)	5.5 μg/mL (2.8–8.7)	5.4 μg/mL^‡^	38%	58%

Trough levels of a subgroup of YP (young patients; < 10 years) (*n* = 46) were routinely measured. Median trough levels (μg/mL), recommended range and clinical remission (%), are shown at 2, 6, 14, 52 and 104 weeks

*Abbreviations*: *IFX* infliximab, *IQR* inter quartile range, *TL* trough level

^†^Clarkston K, Tsai YT, Jackson K, Rosen MJ, Denson LA, Minar P (2019) Development of Infliximab Target Concentrations During Induction in Pediatric Crohn Disease Patients. J Pediatr Gastroenterol Nutr 69:68–74

^‡^van Hoeve K, Dreesen E, Hoffman I, Van Assche G, Ferrante M, Gils A, Vermeire S (2018) Higher Infliximab Trough Levels Are Associated With Better Outcome in Paediatric Patients With Inflammatory Bowel Disease. J Crohns Colitis 12:1316–1325; van Hoeve K, Hoffman I, Vermeire S (2018) Therapeutic drug monitoring of anti-TNF therapy in children with inflammatory bowel disease. Expert Opin Drug Saf 17:185–196

### Predictors of IFX trough levels during IFX treatment

Multivariate analyses of all trough levels (measured both proactively and reactively) of both age groups showed a significant association between higher IFX trough levels and a more intensive treatment regimen, accounted to shorter intervals (*β* − 0.006; 95% CI 0.010 to − 0.001; *p* = 0.011). ATI positivity was negatively associated with IFX trough levels (*β* − 0.681; 95% CI 0.446 to 0.914; *p* < 0.001). Age at the start of therapy (*β* − .013; 95% CI − 0.063 to 0.038; *p* = 0.613) was not significantly associated with IFX trough levels (Table 2).

### Significantly higher risk to develop ATI in YP compared with OP

**Fig. 1:**
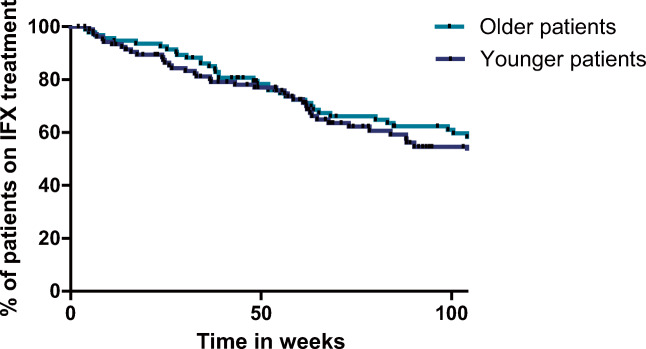
Duration on IFX treatment. Kaplan-Meier survival analysis of duration in days at IFX treatment for YP; young patients < 10 years of age and OP; older patients ≥ 10 years of age (*p* = 0.562)

Taken in account repeated measurement in multivariate analysis, the chance to develop ATI was relatively 0.329 (95% CI 1.2 to 1.01; *p* < 0.001) times lower in OP than YP. In addition, use of immunomodulators reduced the chance to develop ATIs 1.4 (95% CI − 0.31 to 0.39; *p* < 0.001) times more frequently. Despite more immunogenicity in YP during treatment, durability on IFX was not significantly different between the different age groups (*p* = 0.444). Seventy-seven percent of YP were still on IFX treatment after 1 year of follow-up (Fig. 1). This number decreased to 53% after 2 years. Median durability on IFX was 114 weeks in the YP group, compared with 160 weeks in the OP group.

## Discussion

We analysed data of 622 IFX trough levels measured among 110 young (< 10 years) PIBD patients. Moreover, to examine differences in age groups, we compared young to older (≥ 10 years) PIBD patients (105 patients, 295 IFX trough levels). This resulted in a unique overview of clinical efficacy and trough levels of IFX treatment in very young PIBD patients.

In our study, 23% of young PIBD patients had primary nonresponse or secondary loss of response within the first year. This is in line with previous literature, where in 4.5–30% of older PIBD patients, IFX was discontinued [7, 10, 22]. On the other hand, a retrospective study in children ≤ 7 years by Kelsen et al. (*n* = 33, 61% CD; median age at start 5.6; collected between 1999 and 2011) [9] found a very high loss of response to IFX treatment of 64% in their population. This difference in response rate between our study and Kelsen et al. may be explained by an increased use of early TDM in clinical practice over time. The current ECCO ESPGHAN paediatric CD guideline recommends evaluation of IFX dosing preceding the fourth infusion [1]. Early TDM is associated with therapy enhancement and sustained response to IFX and decrease of immunogenicity [14, 19]. In contrast, YP with proactively collected trough levels in our cohort were not significantly more often in clinical remission after 52 weeks.

In 97% of YP, trough levels < 29 μg/mL were found, a cut-off at 2 weeks for predicting treatment success according to Clarkson et al. [17], and at the start of maintenance treatment, suboptimal trough levels (< 5.4 μg/mL) were measured in the majority (72%) of YP patients [18, 19]. Moreover, if we compare the percentage of patients with IFX levels < 3.1 μg/mL in YP (49%), this is almost twice as high as described in previous published litrature of OP (25%) [23].

Furthermore, we found significantly more YP needed treatment optimisation to achieve clinical remission compared with OP. This is in line with the results of both Dotan et al. and Xu et al. who showed a nonlinear correlation between body weight and IFX clearance [3, 4]. De Bruyn et al. [7] showed that patients diagnosed with IBD < 10 years had an increased change of IFX treatment optimisation. However, this did not account for children treated with IFX < 10 years or above. Nonetheless, it is clear that further studies on how to optimize IFX dosing in young PIBD patients are required.

In multivariate analysis, our data showed YP tended to develop ATIs more frequently than OP. In line with other studies in PIBD [3, 6, 13, 14, 24], a positive association between IFX trough levels and shorter interval length was found. As expected, within our cohort of PIBD patients, ATI positivity was associated with lower IFX trough levels. Previous studies demonstrated that low IFX levels were associated with immunogenicity and increased ATI formation [8, 25]. YP in our study who had subtherapeutic trough levels during induction, which could be related to a significantly higher proportion of ATI positive patients. In contrast to recent literature [13, 26], we did not find a correlation between use of immunomodulator (in particular thiopurines) combination therapy and higher trough levels. On the other hand, in line with other studies, our data did show a significant decrease of immunogenicity during combination therapy in YP as well as in OP [27]. PIBD subtype had a nonsignificant effect on IFX trough levels; therefore, it was not possible to further distinguish patients based on disease subtype. We could not determine a direct influence of age on IFX trough levels. This might suggest that it is feasible in YP to reach adequate IFX trough levels, as long as other factors such as immunogenicity can be prevented. As such, it seems even more important that YP are adequately dosed from start of IFX. In addition, we did not find a significant difference in the group of YP receiving proactive TDM, although previous studies showed that frequent proactive TDM could be beneficial to further individualize therapeutic strategies in these patients [6, 28].

Since several other patient factors influence trough levels, it is not possible to give a patient specific dose recommendation. Therefore, the development of a PK model for young patients would be of additional value. Simulation of dosing and treatment schemes based on existing PK data would help to further optimize dosing. Treatment escalation at the start of IFX treatment could lead to supra therapeutic trough levels which might lead to increased immunosuppression or toxicity. However, this has not been described in adult IBD studies, where high target IFX levels (median 10 μg/mL) did not show an increased rate of infections or other adverse events [29, 30]. Besides, high target levels (up to 15 μg/mL) seem not only safe but also beneficial in achieving mucosal healing [29] or remission in (paediatric) IBD patients with perianal fistulising CD [30–33].

Clear strengths of the study were the relatively large number of YP, the number of IFX trough levels (*n* = 622) and a long follow-up period (median 798 days) of this relatively small YP population. However, a limitation of this study is its retrospective design. Data collection and TDM were not performed in a standardized manner (pro- and reactively) which increased the risk for bias. However, a prospective study in this special population will require a very long time. As a consequence of this design, not all patients started with the same dose and were we unable to compare trough levels of YP and OP at the important time point of week 14. Moreover, IFX trough levels and ATI were determined with different assays at the local centres. Comparison of different methodologies in Europe demonstrated significant concordance. Therefore, we estimate this effect to be marginal [34, 35]. Despite concordance between ATI assays, different measurement units made it impossible to compare the effect of ATI levels on a continuous scale [36]. Moreover, if the IFX serum concentration could be detected in a serum sample, ATIs were usually not determined. This may have resulted in a negligible underestimation of the absolute number of ATI positive patients in both groups.

Evaluation of disease activity was limited. PGA was used to evaluate clinical disease activity while endoscopic data and faecal calprotectin levels were missing. Lastly, only 13% of the YP were VEO-IBD patients (< 6 years) which makes it impossible to draw conclusions for this very small but extra vulnerable part of the population in whom IFX is only available for off-label use.

## Conclusion

Our data suggest that up front intensification of the IFX induction scheme in PIBD patients under age of 10 is warranted. Development of a PK model predicting the optimal dose and dosing schemes per patient will be of additional value to confirm and specify our findings. Besides, validation in prospective trials is crucial to further optimize the treatment regimen for young PIBD patients.

## Electronic supplementary material

Online resource 1 Overview of assays and laboratory methods used in different centres (DOCX 16 kb)

Online resource 2 Method of multivariate analysis (DOCX 13 kb)

## Abbreviations

IBD: Inflammatory bowel disease
IFX: Infliximab
OP: Older patients; patients ≥ 10 years of age
PD: Pharmacodynamic
PIBD: Paediatric inflammatory bowel disease
PK: Pharmacokinetic
TDM: Therapeutic drug monitoring
TNF-α: Tumour necrosis factor alpha
VEO-IBD: Very early-onset inflammatory bowel disease
YP: Young patients; patients < 10 years of age
