# Supplementary material for: Infliximab in young paediatric IBD patients: it is all about the dosing
Source: Eur J Pediatr. 2020 Aug 19;179(12):1935–44. doi: 10.1007/s00431-020-03750-0 (PMC7666662; doi:10.1007/s00431-020-03750-0)
Supplement: Supplementary file 1 — Overview of assays and laboratory methods used in different centres (DOCX 16 kb) [file 431_2020_3750_MOESM1_ESM.docx]

**Online resource I – Overview of assays and laboratory methods used in different centres**

|  | Manufacture | Assay trough levels | Assay ATI | Cut-off ATI levels detection |
| --- | --- | --- | --- | --- |
| Erasmus MC | Sanquin, Amsterdam | ELISA  MabTrack Infliximab | Antigen binding test,  MabTrack Infliximab | <12 AE/mL |
| Edmonton Paediatric IBD Clinic | Immunodiagnostik AG, Germany | ELISA | Antigen binding test/ELISA | <2 µg/ml |
| Hôpital Necker-Enfants-Malades | Caltag Laboratories | ELISA | Anti-IFX anti-idiotypic antibodies | <10 AE/mL |
| Our Lady's Children's Hospital | Immune diagnostic | ELISA | ELISA | <10 AE/mL |
| Tampere University Hospital | Promonitor/Sanquin | ELISA  (promonitor) | Antigen binding test (Sanquin) | <12 AE/mL |
| University Hospital Motol | Matrix Biotek | ELISA Q-Inifixi | ELISA Q-ATI | <3 µg/mL |
| Schneider Children's Hospital | Shomron Ben-Horin Sheba Medical Center | ELISA | Anti-lamba based assay chain conjugated antibody | <2.1 µg/mL |
| Dana-Dwek Children’s Hospital | Immunodiagnostik AG, Germany | *IDK*monitor® infliximab drug level ELISA Cat. K 9655 | TNFα blocker ADA,   antibodies against infliximab   ELISA | <10 AE/mL |
| Shaare Zedek Medical Center | Shomron Ben-Horin Sheba Medical Center | ELISA | Anti-lamba based assay chain conjugated antibody | <2.1 µg/mL |
| Universitair Ziekenhuis Gent | apDIA | ELISA | ELISA  (Clone 6B7) | <0.3 µg/mL |
| Amsterdam UMC | Sanquin, Amsterdam | ELISA | Antigen binding test | <12 AE/mL |
| Sheba Medical Center | Shomron Ben-Horin Sheba Medical Center | ELISA | Anti-lamba based assay chain conjugated antibody | <2.1 µg/mL |
| Utrecht Medical Center | Sanquin/LC-MS/MS | ELISA | Antigen binding test | <12 AE/mL |
| Hôpital Robert Debré | Caltag Laboratories | ELISA | Anti-IFX anti-idiotypic antibodies | <10 AE/mL |

**Online resource I. Overview of assays used in different centres.** *Abbreviation; ATI; antibodies-to-infliximab; AE, Arbitrary Units/mL, where 1 AE/mL equals approximately 10 μg/L.*
