# Supplementary material for: Infliximab in young paediatric IBD patients: it is all about the dosing
Source: Eur J Pediatr. 2020 Aug 19;179(12):1935–44. doi: 10.1007/s00431-020-03750-0 (PMC7666662; doi:10.1007/s00431-020-03750-0)
Supplement: Supplementary file 2 — Method of multivariate analysis (DOCX 13 kb) [file 431_2020_3750_MOESM2_ESM.docx]

Online resource 2– Method of multivariate analysis

Both pro- and reactively determined trough levels were included in the models. These data were pooled because all factors potentially influencing the clinical decision to determine levels were included in all multivariate analyses.

First, median trough levels during induction and maintenance treatment were analyzed in a linear mixed model to take into account the repeated measurements. Time was the only fixed effect included in this multivariate analysis. As random effects the intercept was included. Random slopes were tested but not included because it did not significantly improve the model. In the model trough levels were log transformed to satisfy the normality assumption for the error term, evaluated by residual plots.

In a second multivariate analysis all covariates were selected based on clinical relevancy. There was no further selection made for the analysis of covariates. Faecal calprotectin levels were excluded from the multivariate analysis because limited data were available. Fixed effects included in this model were time, sex, age at start, and diagnosis. Variable covariates were; interval (days) between IFX infusions, dose (mg/kg), body surface area (BSA), Clinical Disease Activity (CDA), ATI positivity, Immunomodulator use and laboratory parameters measured simultaneously with the trough levels: C-reactive protein (CRP), Erythrocyte Sedimentation rate (ESR) and albumin. As random effects the intercept was included. Random slopes were tested, but not included because this did not significantly improve the model. In the model trough levels were log transformed to satisfy the normality assumption for the error term, evaluated by residual plots.

Third, to investigate ATI presence over time a multivariate analysis was performed with a Poisson mixed effects model. The covariate time and factors age group and immunomodulator use (time variable) were included in this multivariate analysis. These covariates were included based on clinical relevancy.
